# ZFP36 promotes *VDR* mRNA degradation to facilitate cell death in oral and colonic epithelial cells

**DOI:** 10.1186/s12964-021-00765-4

**Published:** 2021-08-11

**Authors:** Xiangyu Wang, Xuejun Ge, Wang Liao, Yong Cao, Ran Li, Fang Zhang, Bin Zhao, Jie Du

**Affiliations:** 1 Shanxi Province Key Laboratory of Oral Diseases Prevention and New Materials, Shanxi Medical University School and Hospital of Stomatology, No. 63 Xinjian South Road, Taiyuan, 030001 Shanxi China; 2 Department of Child Dental and Preventive Dentistry, Shanxi Medical University School and Hospital of Stomatology, Taiyuan, Shanxi China; 3 Department of Oral Medicine, Shanxi Medical University School and Hospital of Stomatology, No. 56 Xinjian South Road, Taiyuan, 030001 Shanxi China; 4 Department of Endodontics, Shanxi Medical University School and Hospital of Stomatology, Taiyuan, Shanxi China; 5 Department of Cardiology, Hainan General Hospital, Hainan Clinical Medicine Research Institution, Haikou, China; 6 Division of Gastroenterology, Department of Gastroenterology, Shengjing Hospital of China Medical University, Shenyang, Liaoning China; 7 Institute of Biomedical Research, Shanxi Medical University, Taiyuan, Shanxi China

**Keywords:** Vitamin D receptor, Zinc finger protein 36, Oral lichen planus, Inflammatory bowel disease, Y box-binding protein 1

## Abstract

**Background:**

Vitamin D receptor (VDR) plays a vital protective role in oral and colonic epithelial cells. Albeit we know that VDR expression is reduced in the mucosal epithelial layers of autoimmune diseases, the mechanism by which VDR is decreased remains elusive.

**Methods:**

VDR and zinc finger protein 36 (ZFP36) levels in human samples and cell lines were detected by real-time PCR, western blot and immunostaining. Luciferase report assay was used to test cis-elements in *VDR* gene promoter, real-time PCR was applied to measure mRNA decay and western blot was performed to evaluate protein degradation. RNA affinity chromatography assay was used to test protein-mRNA interaction. Co-immunoprecipitation was used to detect protein–protein interaction. The role of ZFP36 in AU-rich elements (AREs) in the 3′ untranslated region (UTR) of *VDR* mRNA was also measured by luciferase report assay.

**Results:**

We identify ZFP36 can bind with the AREs in the 3’UTR of *VDR* mRNA, leading to mRNA degradation in oral and colonic epithelial cells under inflammatory circumstance. Either ZFP36 protein or AREs of *VDR* mRNA mutation abolishes this protein-mRNA binding process. After the key amino acid’s mutation, ZFP36 fails to decrease *VDR* mRNA expression. We also find that VDR physically binds with Y box-binding protein 1 (YBX-1) to block YBX-1’s nuclear translocation and ameliorate cell death in the presence of inflammation.

**Conclusion:**

These findings provide insights into the cause of VDR decrease in oral and colonic epithelial cells under inflammatory condition and explain how VDR maintains cell viability in these cells.

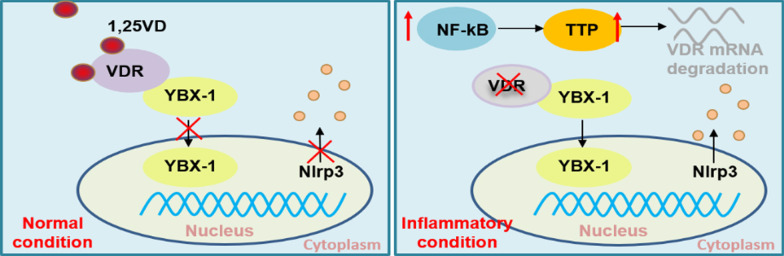

**Video abstract**

**Supplementary Information:**

The online version contains supplementary material available at 10.1186/s12964-021-00765-4.

## Background

The oral or colonic epithelial cells constitute the epithelial barrier that separates harmful materials such as bacteria and toxins in oral cavity or colon lumen [1–3]. Damaged barrier function is capable of resulting in mucosal homeostasis disruption, which triggers or exaggerates mucosal inflammation [4, 5]. Aberrant apoptosis of oral or colonic epithelial cells is regarded as a major pathogenic underpinning resulting in increased epithelial layer permeability and mucosal inflammation [6–8]. Excessive mucosal epithelial cells apoptosis has been indicated in the diseased tissues from patients with oral lichen planus (OLP) and inflammatory bowel disease (IBD), the two typical inflammatory diseases in oral and colonic mucosae [6–8]. Other cell death-associated molecular programmes such as pyroptosis, ferroptosis and necroptosis all contribute to the death of colonic epithelial cells [9]. If these pathways are uncontrollably induced, they may become pathological and lead to inflammatory diseases [9].

Vitamin D receptor (VDR), a nuclear hormone receptor, mediates the biological activity of vitamin D [10]. Upon vitamin D activation, cellular VDR translocates into nucleus and mediates downstream genes expression through binding with the vitamin D response elements (VDREs) in the promoters of target genes [10]. A growing number of epidemiological or experimental studies have reported the close association between VDR deficiency and the development of inflammatory diseases [11–13]. A high prevalence of VDR reductions has been documented in the lesion tissues of auto-immune diseases such as OLP and IBD, especially in the epithelial cells of these inflammation-related diseases [8, 14]. The polymorphisms of *VDR* gene are indicated to be associated with OLP and IBD [15, 16]. These observations imply that VDR expression may be a critical genetic contributor affecting diseases onset or development. In cell and animal studies, genetic interference or deletion of *VDR* exacerbates inflammatory status in oral and colonic epithelia cells while VDR overexpression ameliorates these inflammatory symptoms [14, 17]. In addition, some studies suggest that VDR is a potential therapeutic partner for COVID-19 patients [18]. Vitamin D infusion therapy is proposed to restrain the COVID-19 cytokine storm [19]. Albeit VDR has been claimed to play a key role in suppressing inflammatory responses in a variety of contexts, the molecular mechanism of VDR reduction under inflammatory condition remains a mystery.

As one of the most common regulators of RNA stability, adenylate-uridylate-rich elements (AU-rich elements; AREs) are a region full of adenine and uridine nucleotides in mRNAs in mammalian cells [20]. AREs is thought to harbor a core sequence of AUUUA [21]. Stability of mRNA is controlled by RNA-binding proteins via binding with the AREs located in the 3’ untranslated region (UTR) of mRNAs [22]. Some studies have documented that some proteins (HuA, HuB, HuC, HuD and HuR) bind with AREs to exert stabilizing actions while other proteins (zinc finger protein 36 (ZFP36), AU-binding factor1 (AUF1) and KH-type splicing regulatory protein (KSRP)) have destabilizing effects on target mRNAs [22]. The interaction between AREs and RNA-binding proteins has a prominent influence in the control of gene transcription during immune responses and cell growth and differentiation [20].

Y-box binding protein 1 (YBX-1) is considered to be a versatile protein that embraces a conserved cold-shock domain (CSD), preferentially binds DNA/RNA sequences, and orchestrates transcription [23]. Recent studies have identified that YBX-1 is tightly correlated with Nlrp3 inflammasome which is thought to be a determinant of cell death [24]. Importantly, YBX-1 expression is also increased in various inflammatory diseases [25, 26], indicating upregulation of YBX-1 levels may lead to cell death in inflammatory tissues. Here, we found that the VDR decreases in oral and colonic epithelial cells under inflammatory conditions are due to *VDR* mRNA degradation mediated by ZFP36 induction. The down-regulated VDR expression promotes YBX-1’s nuclear translocation which leads to cell death in oral and colonic epithelial cells.

## Methods and materials

### Human samples collection

Human oral and colonic tissues were obtained from participants at the Stomatological Hospital affiliated with Shanxi Medical University and from active ulcerative colitis patients at Shengjing Hospital of China Medical University respectively as described before [8, 27]. Oral healthy samples were got from individuals amid wisdom teeth extraction. Colonic control specimens were collected from the adjacent normal tissues in each UC patient during endoscopic examination. The study was approved by the Institutional Ethical Committee of both Shanxi Medical University and China Medical University. Written informed consent was signed by each volunteer.

### Cell culture

Human oral keratinocytes (HOKs) were purchased from ScienCell company (Catalog #2610) and cultured in oral keratinocyte medium (OKM) supplemented with 10% FBS and 1% P/S. NCM460 cell line was got from INCELL Corporation (San Antonio, TX) and cultured in M3 medium containing 10% FBS and 1% P/S. To mimic inflammatory condition, cells were treated with 100 ng/ml lipopolysaccharide (LPS, Sigma-Aldrich, O111:B4 *E. coli*) or the culture medium from activated CD4 + T cells for 8 h. The supernatant from CD4 + T cells culture medium accounts for 30% final volumetric concentration. In another experiment, cells were transfected with plasmids for 36 h prior to treatments.

### Oral mucosal epithelia isolation and culture

The entire oral buccal biopsies from human or mice were dissolved into 0.25% dispase II for 12 h, then epithelium and lamina propria layer were directly separated by muscle forceps. Primary mouse oral keratinocytes were cultured as described [28]. Briefly, separated epitheliums were cut into small pieces and digested with 0.25% trypsin (Invitrogen). Single cells were washed and resuspended with OKM supplemented with 1% amphotericin B (SigmaAldrich). Keratinocytes were placed in type-1 collagen-coated dishes.

### CD4 + T cells isolation and stimulation

Peripheral blood samples were collected from patients and treated with Ficoll-Hypaque density gradient centrifugation. CD4 + T cells were isolated using anti-CD4 magnetic particles (BD Biosciences). For stimulation, CD4 + T cells were subjected to anti-CD3 and anti-CD28 antibodies (BD Biosciences Pharmingen) as described before [8].

### Animal colitis models

8-week-old mice were used in this investigation. For the acute TNBS (Sigma-Aldrich)-induced colitis model, 1% TNBS solution was applied to the back skin of mouse for 8 days for pre-sensitization. Overnight-fasted presensitized mice were treated with 100 mg/kg TNBS dissolved in 50% alcohol under anesthesia via intrarectal injection for 3 days, control mice were subjected to 50% alcohol. For the DSS-induced colitis model, mice were treated with 7 days of 2.5% DSS (Fisher Scientific) water and 2 days of tap water.

### Plasmids and transfection

Human and mouse *VDR*, *ZFP36*, *YBX-1*, or *YBX-1ΔC* were amplified and cloned into the pcDNA3.1-HA or pCMV-flag vector. IKKβ plasmids were kindly provided by Yanchun Li (University of Chicago). Fragments from *VDR* promoter or the 3’UTR of *VDR* cDNA were amplified and cloned into the pGL3-Promoter vector (Promega) for luciferase activity detection. The pGL3-VDR-3′UTR-mut was constructed by mutating the ARE sequence 5′ AUUUA3′ to 5′AAAAA3′ in pGL3-VDR-3’UTR plasmid using a QuickChange Site-Directed Mutagenesis Kit (Agilent). ZFP36 point mutations at tyrosine (TAC to GCC) were also performed by this Mutagenesis Kit. Plasmids were transfected into cells with the help of Lipofectamin 3000 (Invitrogen). PCR primers sequences were listed in Additional file 7: Table S1.

### Cell death and viability assays

CytoTox 96 Non-Radioactive Cytotoxicity Assay kit (Promega, Catalog #G1780) was used to measure cell death according to manufacturer’s protocol. Cell viability assay was performed by using the CellTiter-Glo 2.0 kit (Promega, Catalog # G9241).

### IL-1β, IL-18 and caspase 1 measurements

IL-1β and IL-18 concentrations and caspase 1 activity in cell culture media were determined by using specific kits purchased from Abcam according to the manufacturer’s protocols.

### RT-qPCR

Total RNAs from cells or tissues were isolated with TRIzol reagent (Invitrogen), followed by mRNA purification with Dynabeads (Invitrogen). ReverTra Ace Kit (TOYOBO) was selected to synthesize the first-strand cDNA according to the manufacturer’s instructions. SYBR Green Master Mix kit (TOYOBO) was chosen to perform the quantitative polymerase chain reaction using a LightCycler 480 real-time PCR system (Roche). The relative amounts of transcripts were analyzed using the 2^−ΔΔCt^ formula, normalized to GAPDH which was chosen as an internal control. PCR primers sequences were listed in Additional file 7: Table S1.

### Western blot

After ice-cold PBS washes, cells or tissues were lysed in lysis buffer with protease inhibitors (Roche) and heated in boiled water for 5 min. Cell lysates were separated by SDS–PAGE and whole proteins were transferred to a PVDF membrane (Millipore). The membranes were probed with the indicated primary antibodies (1:1000) in cold room overnight, followed by one-hour secondary antibodies incubation at 25 °C. Immunodetection was accomplished by ECL kit (Thermo Fisher Scientific) and visualized with x-ray films. Related antibodies were listed in Additional file 7: Table S2.

### Histology

Mice were anaesthetized prior to sacrifice. Colon tissues were coiled into a ‘Swiss roll’. All fresh human and mouse tissues were fixed with 10% formalin overnight and embedded in paraffin. Tissues were cut into 4 μm for the following staining. For HE examination, slides were stained by hematoxylin and eosin. To localize VDR and ZFP36 expression, slides were incubated with primary antibodies at cold room overnight, followed by one-hour secondary antibodies staining at room temperature. Tissues were then treated with 3,3′-Diaminobenzidine (DAB) and counterstained by hematoxylin.

### mRNA decay assay

Cells cultured in dishes were challenged with LPS or activated CD4 + T cells for 8 h, followed by replacement of fresh media containing 5 μg/ml actinomycin D. After actinomycin D treatment, total cellular RNAs were isolated at different hours for the real-time PCR quantification assay. All mRNA levels were normalized to that at 0 h.

### Protein degradation assay

Cells were transfected with HA-VDR plasmids for 36 h and then challenged with LPS or activated CD4 + T cells for 8 h, followed by replacement of fresh media containing 100 μg/ml cycloheximide (CHX). After CHX treatment, cellular proteins were isolated at different hours for western blot analyses. All protein levels were normalized to that at 0 h.

### Luciferase reporter

Cells were plated onto 24-well plates for culture. At 70% confluence, the cells were transfected with various plasmids (pGL3-VDR-promoter, pGL3-VDR-3’UTR, pGL3-VDR-3’UTR-mut or ZFP36, 500 ng) by using Lipofectamine 3000 as indicated. After 24–32 h, the cells were lysed for luciferase activity measurement with a Bio-Glo Luciferase Assay System kit (Promega). Renilla activity was also detected as an internal control.

### Crosslinking and immunoprecipitation (CLIP)

CLIP assays were performed according to previous studies with some modifications [29]. Briefly, cultured cells were treated with 100 µM 4-thiouridine for 14 h. After washing with PBS, the cells were placed uncovered under UV light at 0.15 J/cm^2^. After irradiation, cells were collected and dissolved in lysis buffer. Supernatants of cell lysates were transferred into new tubes and treated with 1 U/µl RNase T1 at 22 °C for 15 min. 10% lysates were saved for input, the remaining 90% lysates were incubated with antibody-conjugated protein G magnetic beads. After one-hour rotation, the mixture was collected with a magnet followed by a series of washes with IP washing buffer and a high-salt buffer. The mixture was then dissolved into a proteinase K buffer to eliminate proteins. TRIzol Reagent was used to recover input and immunoprecipitated RNAs which were analyzed by real-time PCR.

### RNA affinity chromatography

Biotin-labelled RNA oligonucleotides with AU-rich element or mutated element were denatured at boiled water for 10 min and cooled on ice immediately. 0.4 pmol RNA oligonucleotides were mixed with streptavidin magnetic beads (50 μl, Thermo Fisher Scientific) in binding buffer at 4 °C for 4 h. RNA-beads complexes were then incubated with cell nuclear extract (200 μg) in binding buffer at 4 °C overnight. After washing, RNA–protein mixtures were dissolved in lysis buffer and analyzed by western blot.

### Crypt isolation and colonoid culture

Mouse colonic crypts were isolated as described previously with modifications [30]. Briefly, large intestines were cut longitudinally and flushed with cold PBS. After washing, tissues were cut into ~ 3 mm pieces and resuspended in cold PBS with 10 mM EDTA for 1-h rotation in cold room. Tissues were then shaken fiercely to separate crypts, followed by filtration through a 70-μm strainer. Crypts were gathered by centrifugation at 400 g for 5 min and cultured by using IntestiCult™ Organoid Growth Medium (STEMCELL) according to manufacturer’s instructions. Colonoids were challenged or harvested after 7 days’ culture.

### Chromatin immunoprecipitation (ChIP)

ChIP assays were conducted with a commercial kit (Pierce). In brief, cells were treated with a final concentration of 1% formaldehyde for 10 min, followed by glycine solution treatment (1X) for 5 min in the chemical fume hood. After PBS washes, 100 μl lysis buffer containing protease inhibitors was added to dissolve cells, followed by micrococcal nuclease (10 U/μl) treatments at 37 °C for 15 min. MNase Stop Solution was used to stop the reaction. 10% cell lysates were stored as input samples, the remaining lysates were mixed with anti-p65 or control antibody for rotation at cold room for 12 h. The protein A/G plus agarose beads were then used to precipitate antibody-DNA mixtures. After high salt buffer, low-salt buffer and TE buffer washes, the beads were eluted by elution buffer and the DNAs in the supernatant were collected. Real-time PCR was used to quantify these samples.

### Co-immunoprecipitation (Co-IP)

Co-IP assays were carried out according to Zhang et al. [31]. Briefly, plasmids-transfected cells were lysed by lysis buffer. After centrifugation, supernatants were collected to incubate with antibodies and protein A/G beads (Thermo Fisher Scientific). After four-hour incubation, complexes were subjected to SDS-PAGE for detection.

### NF-κB activity assay

NF-κB activity was determined with NF-κB luciferase reporter kit (BPS Bioscience) according to manufacturer’s instructions. NF-κB luciferase reporter plasmids were transfected into primary cells or cell lines using Lipofectamine 3000 for 36 h. The luciferase activity was tested by a Lumet LB 9507 luminometer.

### Statistical analysis

Data values were shown as means ± SD. Unpaired two-tailed Student’s *t*-test was used for two group comparisons, and one-way analysis of variance (ANOVA) was used for three or more group comparisons. *P* < 0.05 was considered to be statistically significant.

## Results

### VDR expression is downregulated in the oral and colonic epithelial cells under inflammatory condition

**Fig. 1:**
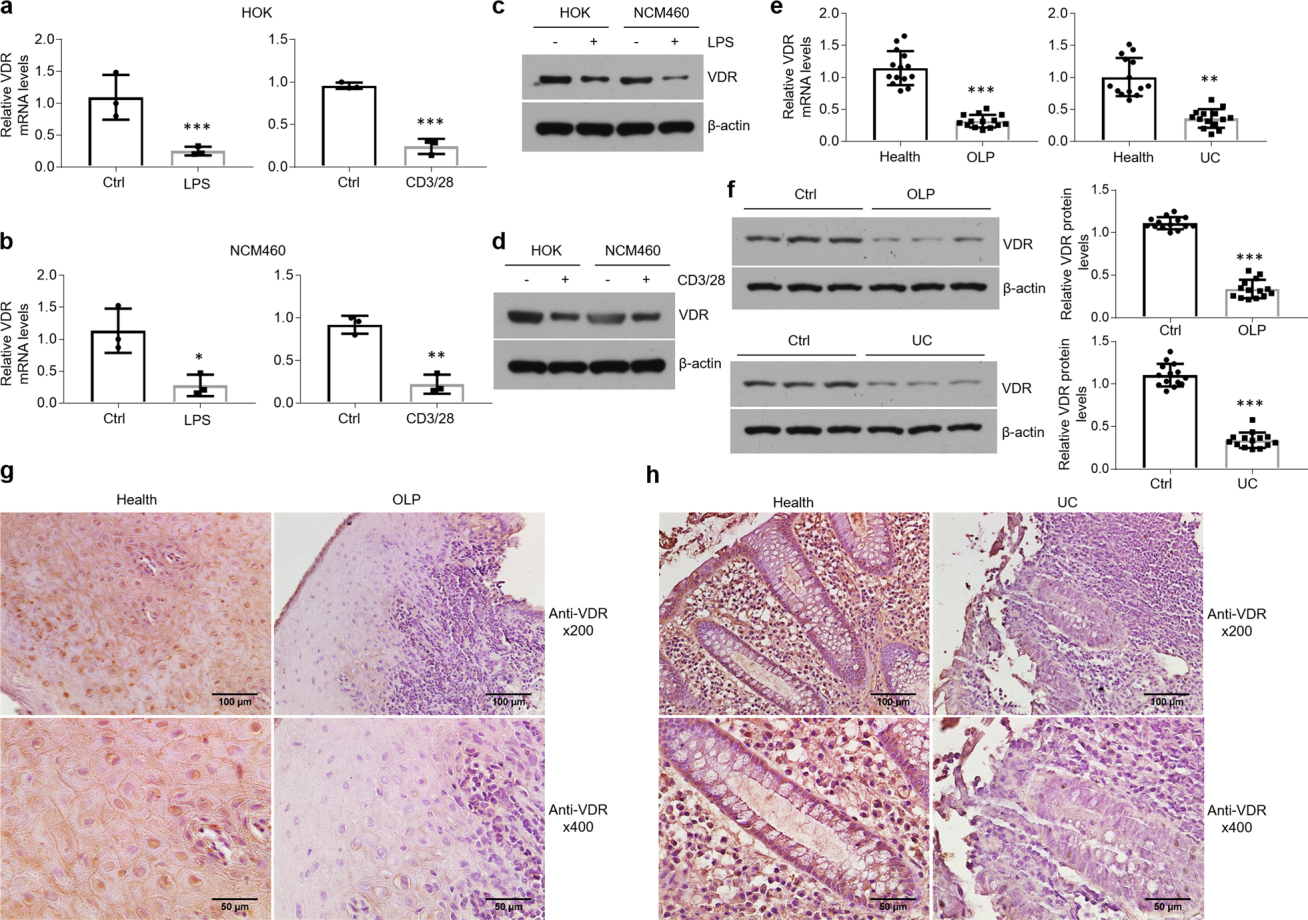
VDR expression is decreased in human oral and colonic epithelial cells in the setting of inflammation. **a**, **b** Real-time PCR quantification of VDR in HOK (**a**) and NCM460 (**b**) cell lines with LPS or activated CD4 + T cells treatment, n = 3. **c**, **d** Western blot analyses of VDR levels in HOK and NCM460 cell lines with LPS (**c**) or activated CD4 + T cells (**d**) challenge, n = 3. **e**, **f** VDR expression in human oral mucosal epitheliums and colonic biopsies assessed by real-time PCR (**e**) and western blot (**f**), n = 14. **g**, **h** Immunostaining showing VDR status in the human oral (**g**) and colonic (**h**) mucosal tissues. *P < 0.05, **P < 0.01, ***P < 0.001 versus corresponding control. Ctrl, control; HOK, human oral keratinocyte; OLP, oral lichen planus; UC, ulcerative colitis

Our previous studies have claimed that VDR functions as a protective mediator in the diseased tissues derived from OLP and IBD [8, 14, 27]. To detect VDR levels in oral and colonic epithelial cells in inflammatory situation, we treated HOKs and NCM460 cells with LPS or the culture medium of activated CD4 + T cells. As shown in Fig. 1, *VDR* mRNA levels were decreased in the presence of LPS or culture medium from activated CD4 + T cells in human cell lines (Fig. 1a, b). We then established primary mouse oral keratinocytes and colonoids culture models, and found that *VDR* mRNA expression was also downregulated after treatments (Additional files 1 and 2: Figure S1a, b and S2a). Western blot data showed that VDR protein levels were decreased as well following treatments (Fig. 1c, d and Additional file 1: Figure S1c, d). In accordant with these data in vitro, both mRNA and protein levels of VDR exhibited reductions in the human diseased samples compared to healthy controls (Fig. 1e, f). Immunostaining results demonstrated that VDR expressed in control epithelial cells was not found in the lesion tissues (Fig. 1g, h). We then established colitis animal models using TNBS and DSS (Additional file 1: Figure S1e, f), and severe mucosal ulceration was observed upon either TNBS or DSS treatment (Additional file 1: Figure S1g). Consistently, VDR expression showed a decrease in the mucosal epithelial cells of colitis (Additional file 1: Figure S1h). The animal models of OLP are not involved in this study.

### VDR mRNA degradation contributes to down-regulation of VDR expression following LPS or culture medium of activated CD4 + T cells challenge

**Fig. 2:**
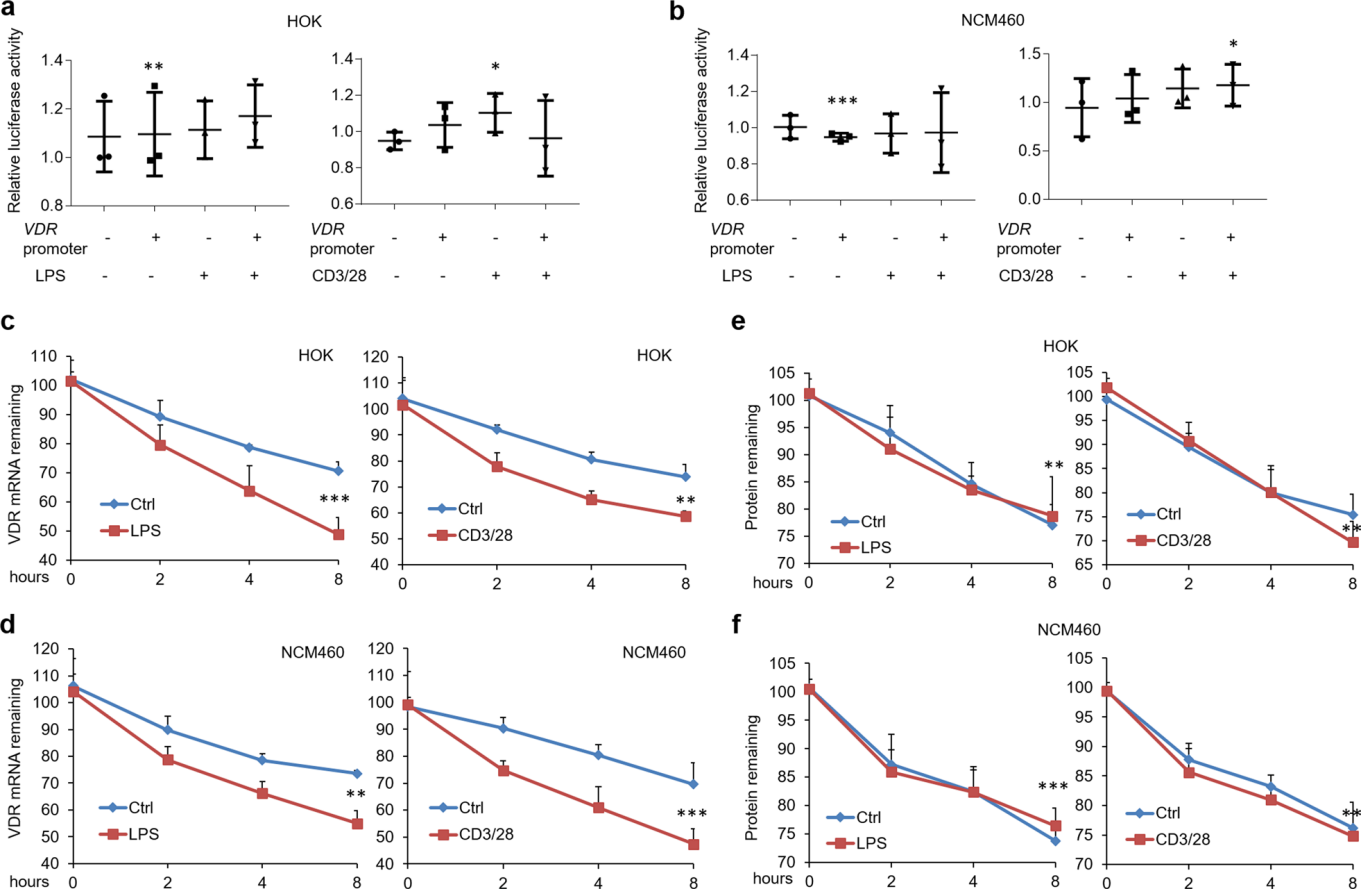
Rapid *VDR* mRNA decay is found in human oral and colonic epithelial cells following treatments. **a**, **b** Luciferase activity in HOK (**a**) and NCM460 (**b**) cell lines transfected with pGL3-promoter plasmids containing the promoter region of human *VDR* gene. **c**, **d** *VDR* mRNA decay assays in HOK (**c**) and NCM460 (**d**) cell lines following LPS or activated CD4 + T cells treatment. **e**, **f** Protein degradation assays of HOK (**e**) and NCM460 (**f**) cell lines following treatments. n = 3 each group, *P < 0.05, **P < 0.01, ***P < 0.001 versus corresponding control. HOK, human oral keratinocyte

To dissect the molecular mechanism of VDR reduction, the transcription levels of *VDR* gene were examined at first. We inserted the promoter region (− 3000 to 0 bp) of human *VDR* gene into pGL3-promoter plasmids and performed luciferase report assay. As shown in Fig. 2, the luciferase activities were not affected in the setting of inflammation (Fig. 2a, b), implying VDR decrease is unlikely due to the dysregulation of transcription level. We then detect the post-transcriptional regulation by monitoring *VDR* mRNA decay. Importantly, *VDR* mRNA was rapidly degraded following LPS or culture medium of activated CD4 + T cells challenge in human cell lines (Fig. 2c, d), and so was that in primary epithelial cells from mouse (Additional file 2: Figure S2b, c). Finally, we tested VDR protein degradation in HA-tagged VDR plasmids-transfected cells after cycloheximide treatment. Our data showed that VDR protein was not influenced in the setting of inflammation (Fig. 2e, f and Additional file 2: Figure S2d). These observations suggest that the reductions of VDR levels are unlikely due to the mediation on gene transcription or protein stability, and instead likely due to accelerated mRNA decay.

### ZFP36 is identified as the key determinant for VDR mRNA degradation

**Fig. 3:**
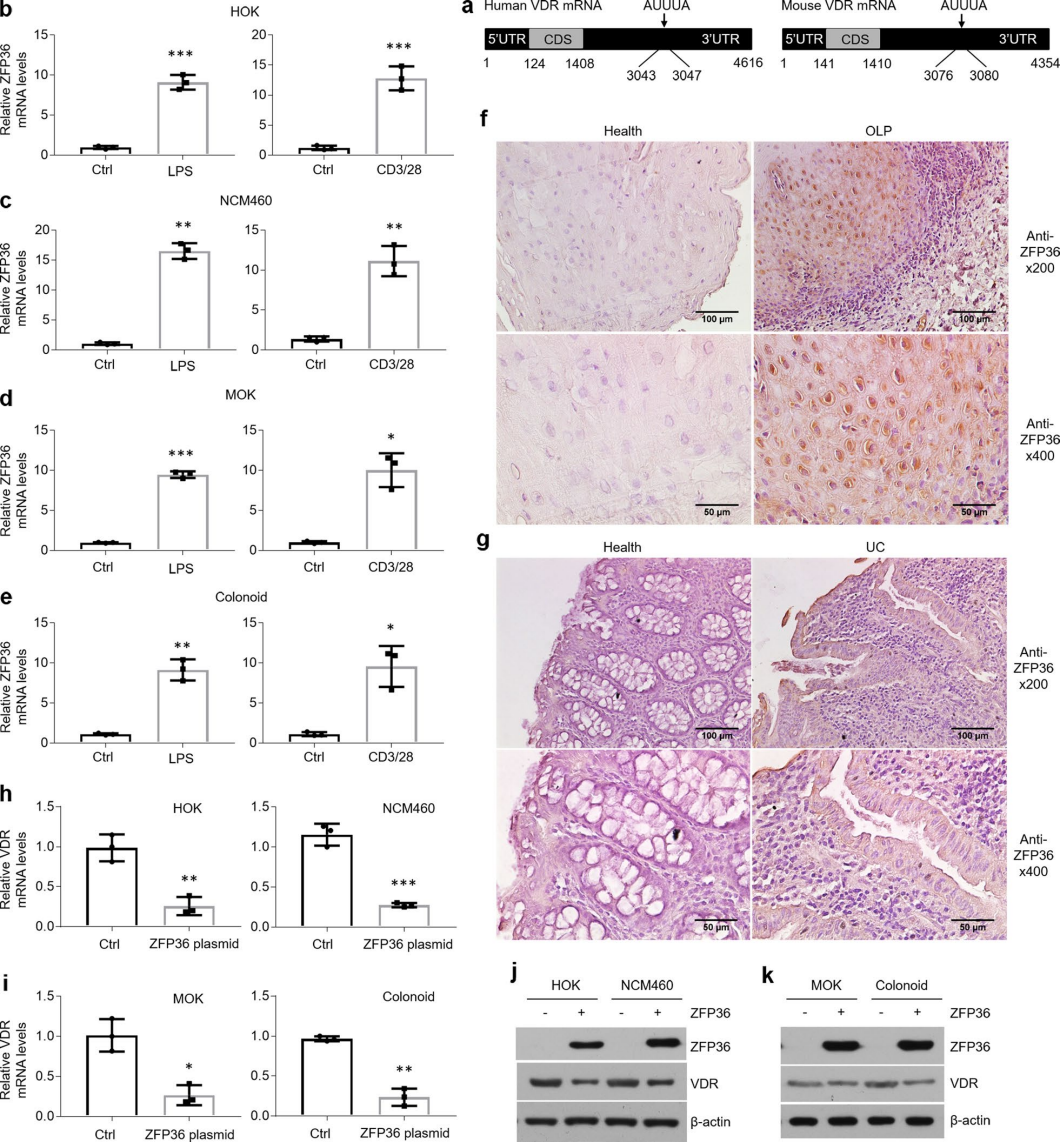
ZFP36 is upregulated in oral and colonic epithelial cells under inflammatory circumstance. **a** Schematic illustration of AREs in *VDR* mRNAs. **b**–**e** Real-time PCR quantification of ZFP36 in HOKs (**b**), NCM460 (**c**), MOKs (**d**) and colonoids (**e**) following LPS or activated CD4 + T cells treatment. **f**, **g** Immunostaining showing ZFP36 status in the human oral (**f**) and colonic (**g**) mucosal tissues. **h**, **i** *VDR* mRNA levels in human (**h**) and mouse (**i**) epithelial cells with ZFP36 overexpression. **j**, **k** Western blot detections of human (**j**) and mouse (**k**) epithelial cells with ZFP36 plasmids transfection. n = 3 each group, *P < 0.05, **P < 0.01, ***P < 0.001 versus corresponding control. Ctrl, control; HOK, human oral keratinocyte; MOK, mouse oral keratinocyte

AU-rich elements in the 3’UTR of mRNAs are considered to control mRNAs’ decay [20, 21, 32]. We therefore speculated that there are AREs located in the 3’UTR of *VDR* mRNA. Consistent with our hypothesis, the core AUUUA sequence was found in human or mouse *VDR* mRNA (Fig. 3a). After quantitative PCR screening, most of AREs-associated binding proteins were not induced upon treatments (Data not shown) except for ZFP36 (Fig. 3b, e). Moreover, increased ZFP36 expression was also observed in the epithelial cells of diseased tissues derived from human or mouse by immunostaining (Fig. 3f, g and Additional file 3: Figure S3a). To confirm the function of ZFP36 in VDR decrease, we transfected ZFP36 plasmids into human and mouse epithelial cells and discovered that VDR mRNA and protein levels showed robust reductions in response to ZFP36 overexpression (Fig. 3h, k).

To explore the molecular underpinning by which ZFP36 is overproduced in the setting of inflammation, we screened the promoter region of *ZFP36* and found NF-κB binding site (Additional file 3: Figure S3b). In support of this finding, we confirmed NF-κB activities were remarkably enhanced in epithelial cells following treatments and verified that NF-κB p65 could bind with κB element in the promoter of *ZFP36* gene by ChIP assay (Additional file 3: Figure S3c, d). IKKβ overexpression, which is thought to activate NF-κB pathway, increased ZFP36 status in oral and colonic epithelial cells (Additional file 3: Figure S3e, f).

### ZFP36 exerts its suppressive action by binding with the AREs in the 3’UTR of VDR mRNA

**Fig. 4:**
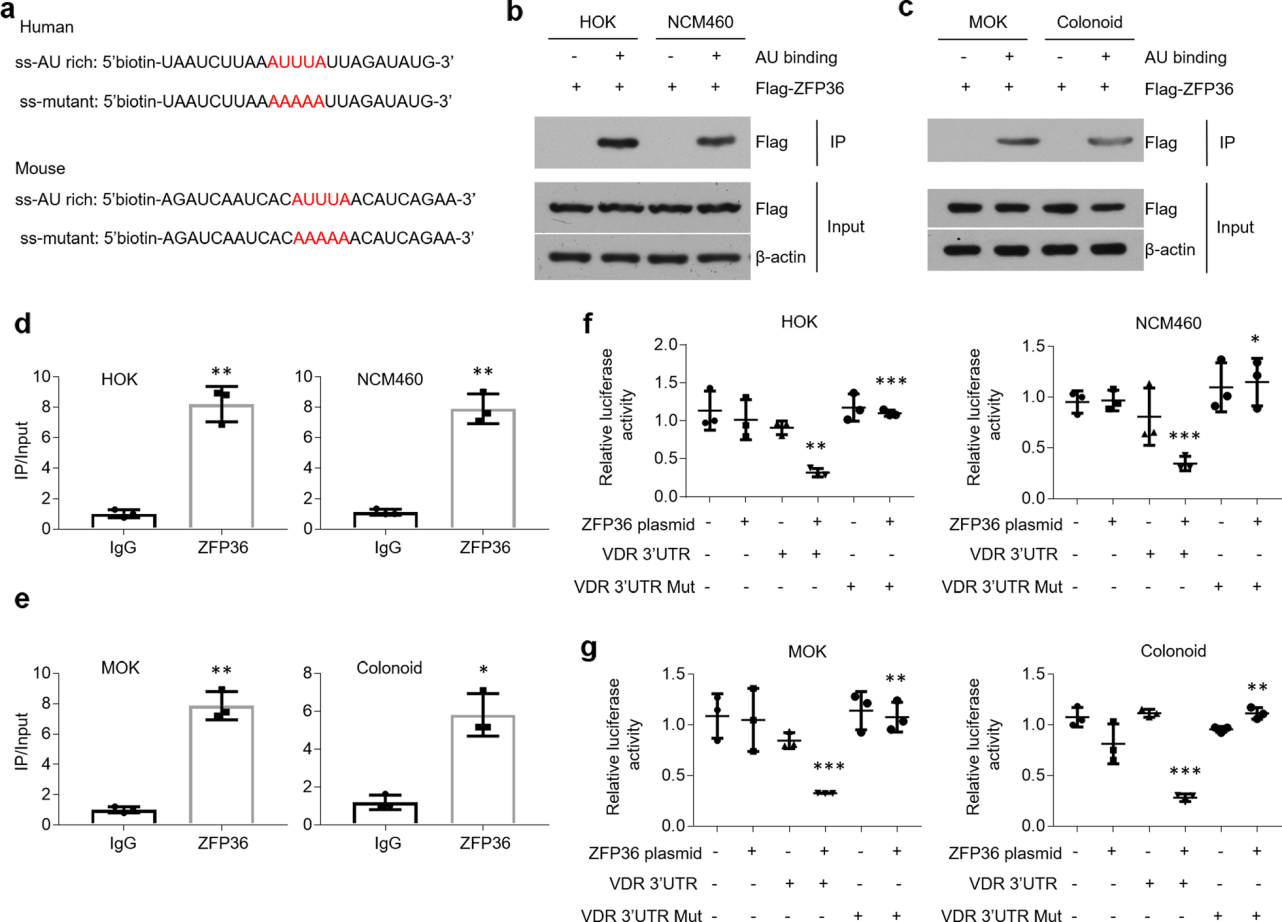
ZFP36 binds with AREs in the 3’UTR of *VDR* mRNA. **a** ssRNA probes with AU-rich core sequence or mutated sequence. **b**, **c** RNA probes pull-down and western blot examinations of cell lysates from human (**b**) and mouse (**c**) cells transfected with ZFP36 plasmids. **d**, **e** CLIP assays against ZFP36 or control IgG antibodies showing ZFP36 protein selectively interacts with AREs in the 3’UTR of *VDR* mRNA in human (**d**) and mouse (**e**) cells. **f**, **g** Luciferase report assessments of human (**f**) and mouse (**g**) cells transfected with ZFP36 plasmids and pGL3-promoter plasmids containing AREs (indicated as VDR 3’UTR) or mutated AREs (indicated as VDR 3’UTR Mut) sequences. n = 3 each group, *P < 0.05, **P < 0.01, ***P < 0.001 versus corresponding control. HOK, human oral keratinocyte; MOK, mouse oral keratinocyte

Although the increase of ZFP36 had been mentioned above, the interaction between ZFP36 and AREs in the 3’UTR of *VDR* mRNA should be verified. We synthesized biotin-labelled RNA nucleotides flanking AREs in 3’UTR of *VDR* mRNA (Fig. 4a). In RNA pull-down assays, ZFP36 was immunoprecipitated by AU-rich RNA nucleotides (Fig. 4b, c). CLIP data showed that ZFP36 selectively interacted with the AU-rich RNA nucleotides with a six–eightfold higher affinity compared with control groups (Fig. 4d, e). The suppressive role of ZFP36 in *VDR* mRNA was also confirmed by performing luciferase activity assays (Fig. 4f, g). After mutation on the core sequence of RNA nucleotides (Fig. 4a), ZFP36 failed to bind with mutated nucleotides and the repressive effect was lost compared to non-mutated controls (Additional file 4: Figure S4a and 4f, g). Previous studies have suggested that Tyr^151^ in human ZFP36 protein plays a key role in the binding process [33]. We then mutated Tyr^151^ and Tyr^143^ to alanine in human and mouse ZFP36 proteins respectively, and found that AU-rich RNA nucleotides lost the ability of precipitating ZFP36 (Additional file 4: Figure S4b, c). Accordantly, ZFP36 mutation could not decrease *VDR* mRNA expression in oral and colonic epithelial cells (Additional file 4: Figure S4d, e).

### VDR inhibits YBX-1 nuclear translocation via physical interaction

**Fig. 5:**
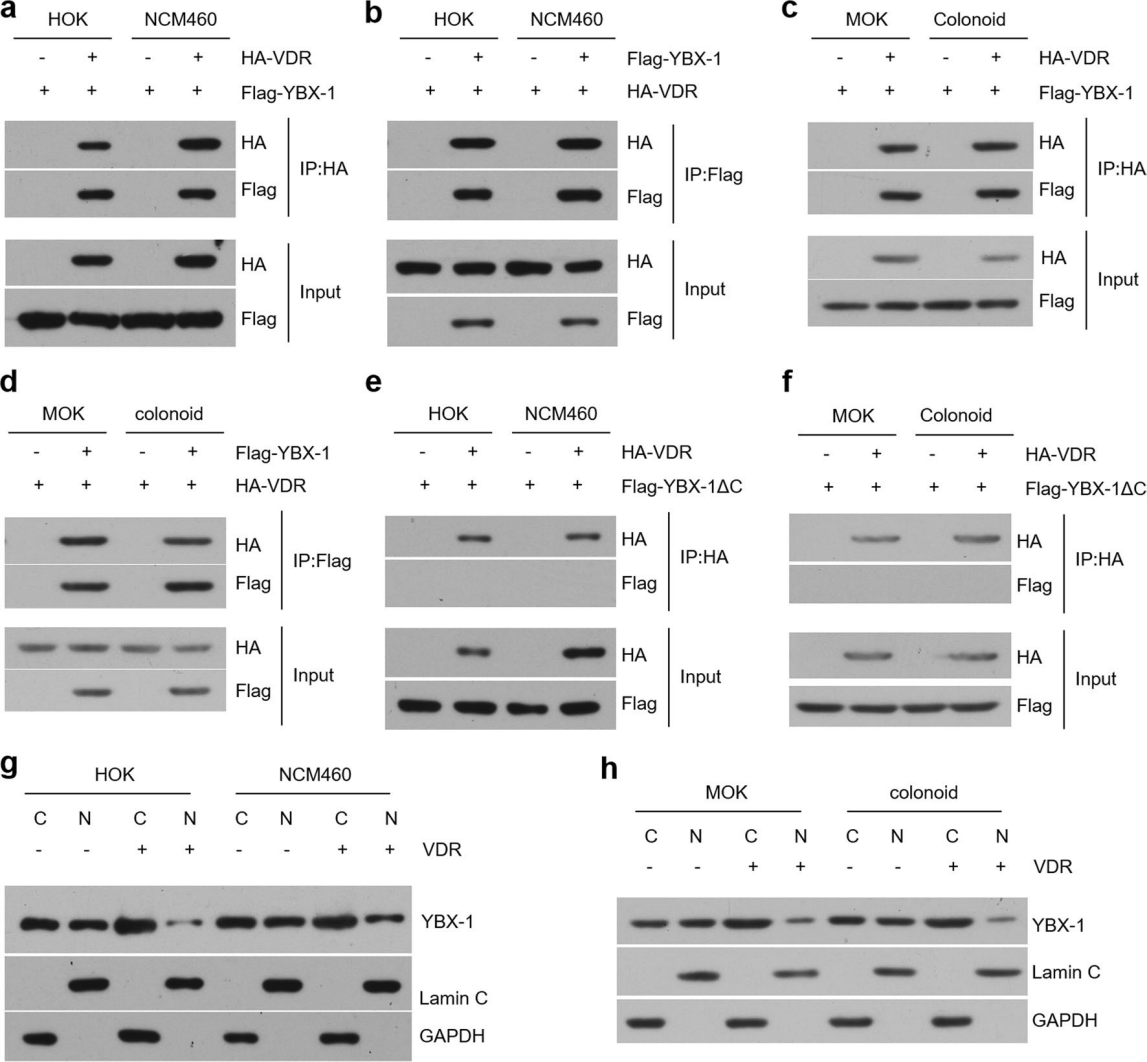
VDR physically interacts with YBX-1 in oral and colonic epithelial cells. **a**, **b** Co-IP and western blot analyses of HOK and NCM460 cell lines transfected with YBX-1 and VDR plasmids. Precipitation was performed by using HA (**a**) or Flag (**b**) antibodies. **c**, **d** Co-IP and western blot detections of cells overexpressing YBX-1 and VDR. Precipitation was performed by using HA (**c**) or Flag (**d**) antibodies as shown. **e**, **f** Western blot analyses of human (**e**) and mouse (**f**) cells transfected with VDR or YBX-1ΔC plasmids. **g**, **h** Human (**g**) and mouse (**h**) cells were transfected with or without VDR plasmids. Nuclear (N) and cytoplasmic (C) fractions were extracted and determined by western blotting. Lamin C is a nuclear marker, and GAPDH is a cytoplasmic marker

VDR is predicted to bind with YBX-1 which induces inflammasome through activating Nlrp3 [24, 34]. To confirm this prediction, we co-transfected VDR and YBX-1 plasmids into epithelial cells and observed the physical interaction between these two proteins (Fig. 5a–d). Since the C-terminal domain of YBX-1 has an effect on protein–protein interaction (Additional file 5: Figure S5a), we constructed ΔC plasmids coding YBX-1 proteins without C-terminal domain (Additional file 1: Figure S5b). As shown in Fig. 5, after ΔC plasmids transfection, the interaction between VDR and YBX-1 proteins was not observed (Fig. 5e, f). Based on these immunoprecipitation results, we reasoned that VDR might bind with YBX-1 to preclude its nuclear translocation. As expected, YBX-1 expression in the nucleus was largely reduced following VDR overexpression compared to control groups (Fig. 5g, h).

### VDR interacts with YBX-1 to inhibit cell death in oral and colonic epithelial cells

**Fig. 6:**
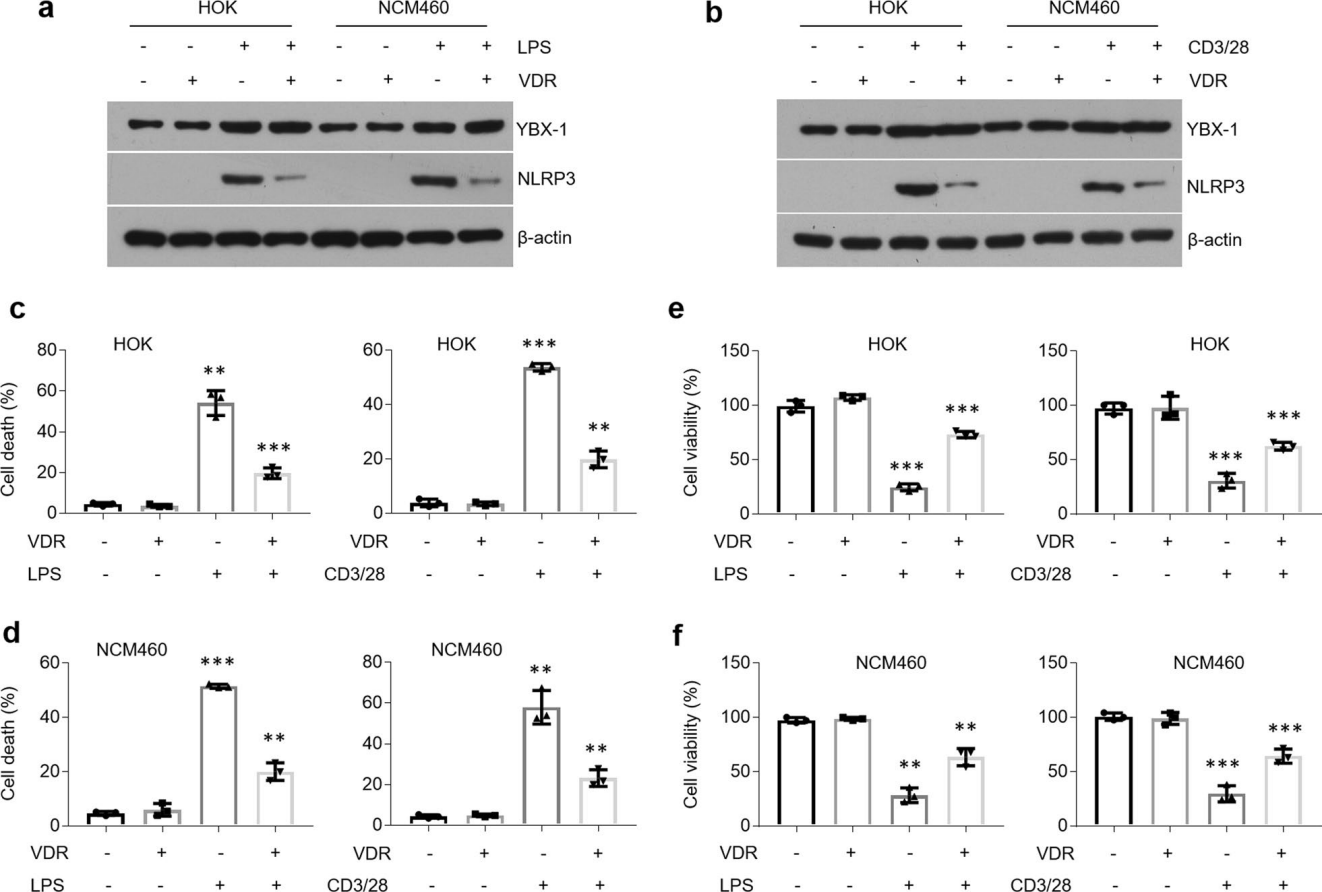
VDR reverses cell death in oral and colonic epithelial cells following treatments. **a**, **b** Western blot showing YBX-1 and Nlrp3 levels in HOK and NCM460 cell lines overexpressing VDR with or without LPS (**a**) or activated CD4 + T cells (**b**) treatment. **c**, **d** Cell death measurements in HOK (**c**) and NCM460 (**d**) cell lines transfected with VDR plasmids with or without LPS or activated CD4 + T cells treatment. **e**, **f** Cell viability detections in HOK (**e**) and NCM460 (**f**) cell lines transfected with VDR plasmids in the presence or absence of LPS or activated CD4 + T cells treatment. n = 3 each group, *P < 0.05, **P < 0.01, ***P < 0.001 versus corresponding control. HOK, human oral keratinocyte

Previous studies have demonstrated that YBX-1 induces Nlrp3-infammosome and caspase 1 overproductions [24]. In this investigation, we noticed VDR reversed the elevated levels of Nlrp3 and caspase 1 activity following LPS or activated CD4 + cells treatment without affecting YBX-1 expression (Fig. 6a, b and Additional file 6: Figure S6a, b). Caspase 1 directly cleaves the precursor cytokines pro-IL-1β and pro-IL-18 into IL-1β and IL-18 [35, 36]. Concomitantly, overproductions of IL-1β and IL-18 were compromised in the presence of VDR plasmids (Additional file 6: Figure S6c–f). Active caspase 1 also triggers a kind of programmed cell death known as pyroptosis [35]. As expected, cell death was ameliorated by VDR overexpression following treatments while cell viability was improved (Fig. 6c–f).

## Discussion

Vitamin D-VDR system is famous for its functions in calcium absorption, bone homeostasis, innate and adaptive immunity and cellular growth and apoptosis [37]. VDR, which is activated by 1,25(OH)_2_D_3_, leads to alterations in the expression of numerous genes in a variety of human tissues [37]. VDR plays a vital role in maintaining the epithelial layer homeostasis in oral and colonic tissues which are exposed to various microorganisms directly [8, 14]. Previous investigations have suggested that VDR expression is compromised in the mucosal epithelial cells of autoimmune diseases such as OLP and IBD [13, 14]. Consistently, in this study, we treated oral and colonic epithelial cells with LPS or the culture medium of activated CD4 + T cells and confirmed that VDR expression is decreased in these cells following treatments. Since VDR is known as a nuclear receptor, the immunostaining data showed that the brown positive signals are mainly expressed in the nucleus instead of cytoplasm. Some studies have emphasized that Vitamin D and its nuclear receptor mediate innate and adaptive immune response in the gut [37, 38]. Indeed, VDR levels in the lamina propria regions seem to be down-regulated as well in the lesion tissues (Fig. 1), indicating VDR might be required for controlling inflammatory responses in immune cells.

The molecular mechanism by which VDR expression is down-regulated in oral and epithelial cells remains poorly understood. Some studies report that miR-346 takes a toll on VDR expression by targeting its mRNA [39], but gene translation and protein degradation are not explained. Here, we inserted the promoter region of *VDR* gene into pGL3-promoter plasmids and luciferase activity results showed that LPS or activated CD4 + T cells challenge can’t affect VDR expression at gene transcription level. We then transfected HA-tagged VDR plasmids into cells to verify that protein degradation dose not contribute to the decreases of VDR levels after treatments. RNA instability is the only determinant for VDR decreases in oral and colonic epithelial cells in the presence of treatments.

AU-rich element is one of the most common regulators of RNA stability in mammalian cells [20, 21]. We found that both human and mouse *VDR* mRNAs contain AREs in the 3’UTR regions, suggesting AREs-associated proteins may interact with *VDR* mRNA leading to RNA degradation. ZFP36, one of the proteins that bind with AREs, is upregulated in the oral and colonic epithelial cells following treatments. This finding is consistent with other studies reporting ZFP36 is induced by LPS in THP-1 cells [40]. We then verified the interaction between ZFP36 protein and AREs in the 3’UTR of *VDR* mRNA through a set of biological experiments and confirmed that ZFP36 overexpression in oral and colonic epithelial cells decreases VDR levels. Since ZFP36 is mainly upregulated in the epithelial cells of human biopsies rather than lamina propria (Fig. 3f, g), we cannot draw a conclusion that the reductions of VDR in lamina propria are also due to instability of mRNAs.

YBX-1 is able to activate *Nlrp3* mRNA transcription to produce excessive inflammasome and trigger caspase 1-dependent cell death [35, 36]. In line with previous proteomics analysis [34], we found that VDR physically interacts with YBX-1, the event that blocks YBX-1’s nuclear translocation and the following programmed cell death. This finding elucidates the mechanism by which VDR maintains epithelial cells homeostasis.

Although the relationship between IBD and OLP is not fully explained, IBD patients have been reported to develop OLP after anti-TNF-α treatments, demonstrating the occurrence of OLP may be a side effect of the use of TNF-a inhibitors [41]. How to manage these diseases efficaciously remains a problem. While diverse therapies for ameliorating the clinical symptoms of OLP and IBD are under development or in the clinic, long-term therapeutic efficacy continues to be a serious challenge [42–44].

## Conclusion

**Fig. 7:**
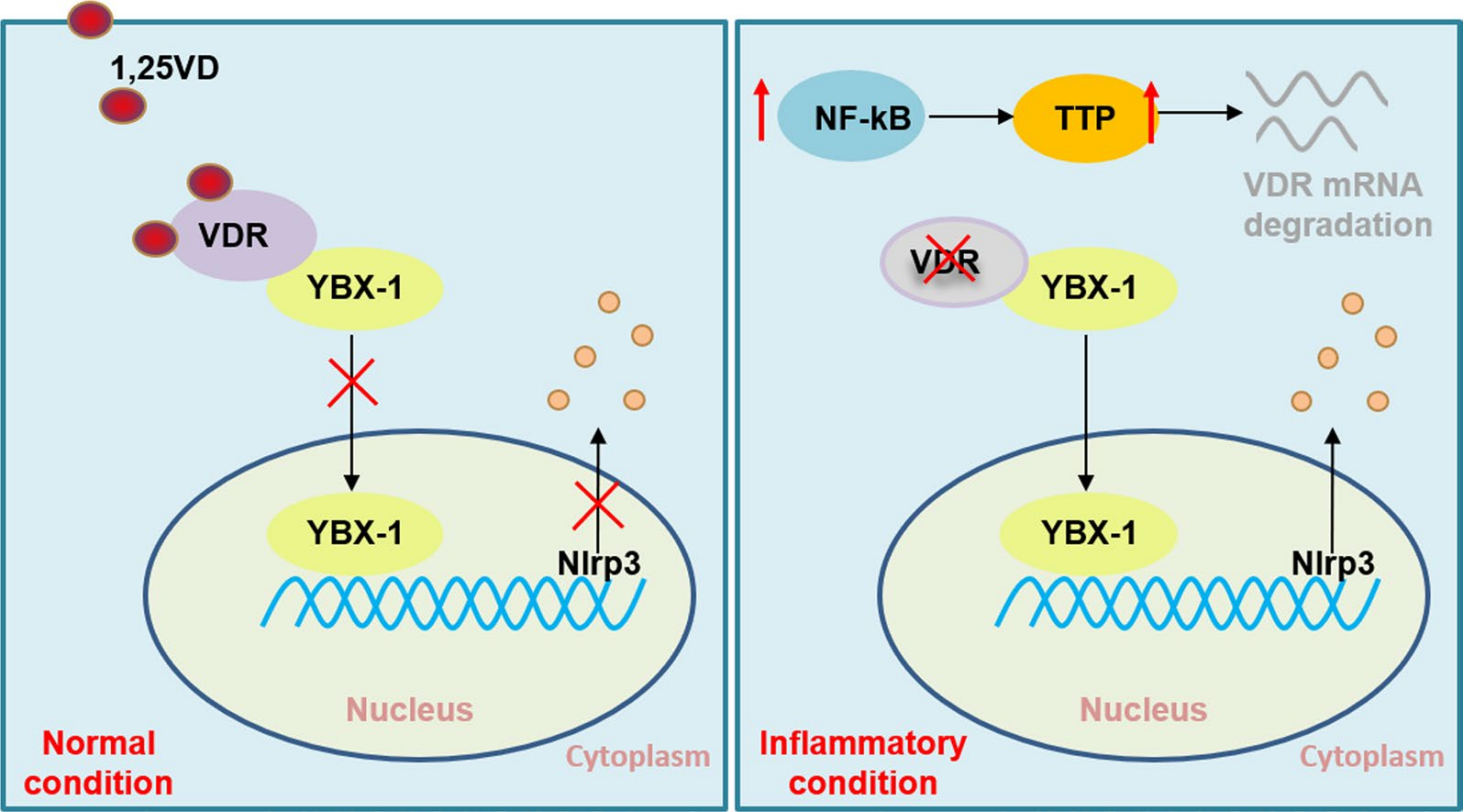
Schematic of the mechanism of VDR decreases and its effect on cell death

In this study, we present evidence that VDR decrease in oral and colonic epithelial cells under inflammatory condition is due to ZFP36-induced mRNA instability, ruling out the possibility that VDR levels are reduced at gene transcription and protein levels. The deficiency of VDR in these epithelial cells results in cell death by releasing YBX-1 into nucleus (Fig. 7). By focusing on the molecular underpinning of VDR decrease, we uncover a novel therapeutic approach to suppress cell death via targeting ZFP36 in oral and colonic epithelial cells. This strategy emphasizes the power of RNA stability, tackling the problem at the post-transcriptional level. Exploiting the power of VDR and its mRNA stability offers a very unusual way in the treatment of OLP and IBD.

## Supplementary Information

**Additional file 1.Supplemental figure 1.** VDR levels are decreased in mouse oral and colonic epithelial cells under inflammation condition. (a and b) Real-time PCR quantification of VDR in mouse primary oral (a) and colonic (b) epithelial cells with LPS or activated CD4+ T cells treatment, n = 3. (c and d) Western blot determinations of VDR levels in mouse primary oral and colonic epithelial cells following LPS (c) or activated CD4+ T cells (d) challenge, n = 3. (e and f) Schematic illustration of TNBS (e) or DSS (f) treatment protocol. (g) HE stained-colonic sections from control and TNBS- or DSS-treated mice. (h) Immunostaining showing VDR expression in the colonic epithelial cells of TNBS- or DSS-treated mice. *P < 0.05, **P < 0.01, ***P < 0.001 versus corresponding control. Ctrl, control; MOK, mouse oral keratinocyte.

**Additional file 2.Supplemental figure 2.** VDR mRNA degradation is observed in mouse oral and colonic epithelial cells following treatments. (a) Colonoid cultured from mouse colonic crypts. (b and c) VDR mRNA decay in MOKs (b) and colonoids (c) following LPS or activated CD4+ T cells treatment. (d) Western blot determinations of HOK and NCM460 cell lines transfected with HA-VDR plasmids. n = 3 each group, *P < 0.05, **P < 0.01, ***P < 0.001 versus corresponding control. MOK, mouse oral keratinocyte.

**Additional file 3.Supplemental figure 3.** ZFP36 is induced by activated NF-κB pathway. (a) Immunostaining analyses of ZFP36 expression in the colonic mucosal tissues from TNBS- or DSS-treated mice. (b) Schematic illustration of NF-κB binding sites in the promoter region of ZFP36 gene. (c) NF-κB activity measurements in HOK, NCM460, MOK and colonoid following treatments as shown. (d) ChIP assays against NF-κB p65 antibody in epithelial cells as indicated. (e and f) Western blot detections of human (e) and mouse (f) epithelial cells with IKKβ plasmids transfection. n = 3 each group, *P < 0.05, **P < 0.01, ***P < 0.001 versus corresponding control. Ctrl, control; HOK, human oral keratinocyte; MOK, mouse oral keratinocyte.

**Additional file 4.Supplemental figure 4.** Mutated ZFP36 fails to bind with AREs in the 3’UTR of VDR mRNA. (a) Mutated-RNA probes pull-down and western blot examinations of cell lysates from cells transfected with ZFP36 plasmids. (b and c) RNA probes pull-down and western blot determinations of cell lysates from human (b) and mouse (c) cells transfected with ZFP36 mutated plasmids as shown. (d and e) Real-time PCR quantification of VDR mRNA levels in human (d) and mouse (e) cells transfected with ZFP36 mutated plasmids. n = 3 each group, *P < 0.05, **P < 0.01, ***P < 0.001 versus corresponding control. Ctrl, control; HOK, human oral keratinocyte; MOK, mouse oral keratinocyte.

**Additional file 5.Supplemental figure 5.** Construction of YBX-1ΔC plasmids. (a) Schematic structures of YBX-1 proteins. (b) The sketch map of plasmids.

**Additional file 6.Supplemental figure 6.** The enhanced caspase 1, IL-1β and IL-18 levels are relieved by VDR overexpression following treatments. (a and b) Caspase 1 activity assessments of HOK (a) and NCM460 (b) cell lines. (c and d) IL-1β concentrations in HOK (c) and NCM460 (d) cell lines. (e and f) IL-18 concentrations in HOK (e) and NCM460 (f) cell lines. Cells were transfected with empty or VDR plasmids following LPS or activated CD4+ T cells treatment. n = 3 each group, *P < 0.05, **P < 0.01, ***P < 0.001 versus corresponding control. HOK, human oral keratinocyte.

**Additional file 7.** Primers and antibodies information.

## Data Availability

These are part of the additional figures.

## Abbreviations

AREs: AU-rich elements
HOK: Human oral keratinocyte
IBD: Inflammatory bowel disease
IKKβ: IκB kinase β
LPS: Lipopolysaccharide
OLP: Oral lichen planus
VDR: Vitamin D receptor
VDREs: Vitamin D response elements
YBX-1: Y box-binding protein 1
ZFP36: Zinc finger protein 36
