# Supplementary figures and images for: ZFP36 promotes VDR mRNA degradation to facilitate cell death in oral and colonic epithelial cells
Source: Cell Commun Signal. 2021 Aug 11;19:85. doi: 10.1186/s12964-021-00765-4 (PMC8355874; doi:10.1186/s12964-021-00765-4)

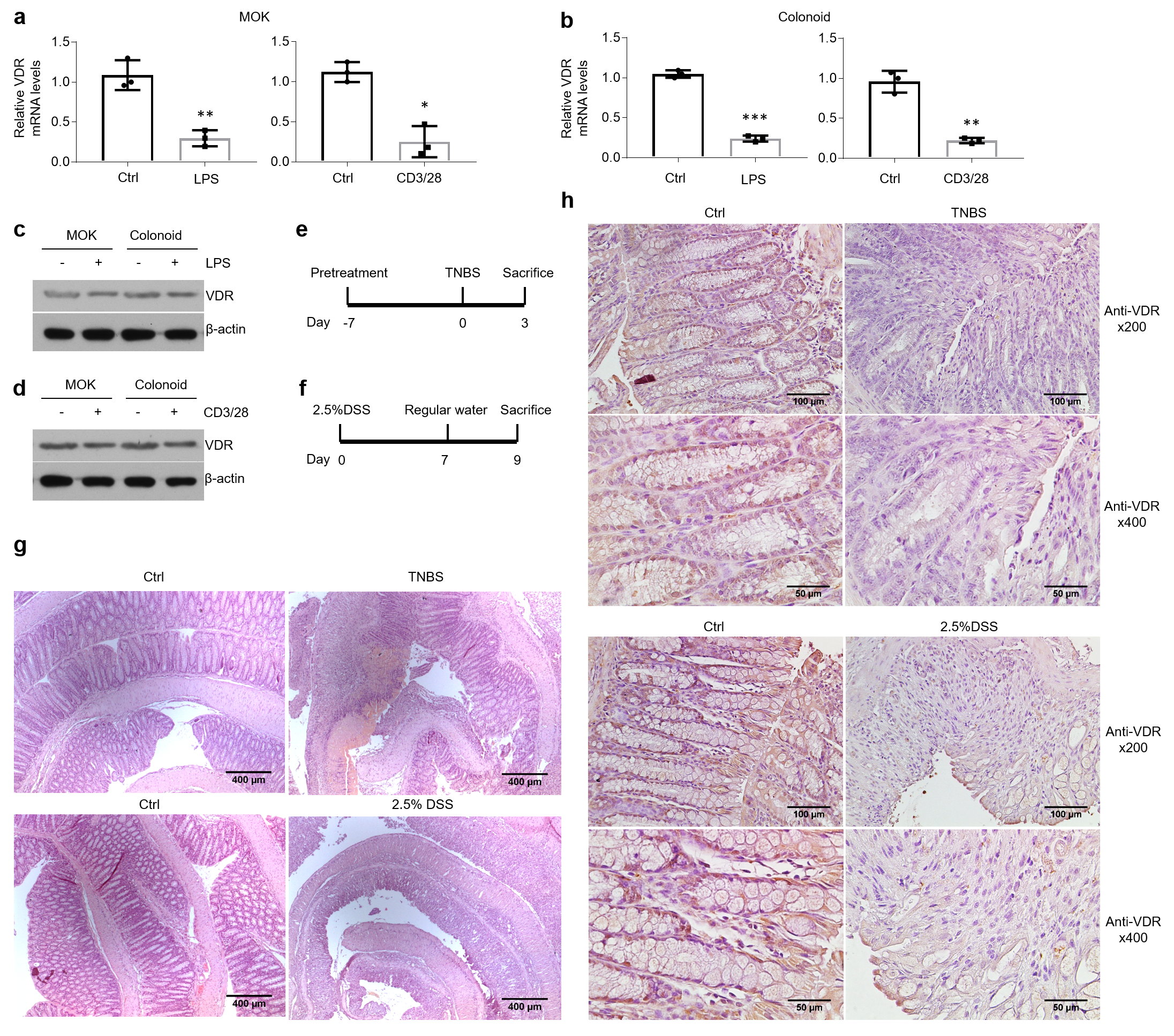

Supplement: Supplementary file 2 — Additional file 1.Supplemental figure 1. VDR levels are decreased in mouse oral and colonic epithelial cells under inflammation condition. (a and b) Real-time PCR quantification of VDR in mouse primary oral (a) and colonic (b) epithelial cells with LPS or activated CD4+ T cells treatment, n = 3. (c and d) Western blot determinations of VDR levels in mouse primary oral and colonic epithelial cells following LPS (c) or activated CD4+ T cells (d) challenge, n = 3. (e and f) Schematic illustration of TNBS (e) or DSS (f) treatment protocol. (g) HE stained-colonic sections from control and TNBS- or DSS-treated mice. (h) Immunostaining showing VDR expression in the colonic epithelial cells of TNBS- or DSS-treated mice. *P < 0.05, **P < 0.01, ***P < 0.001 versus corresponding control. Ctrl, control; MOK, mouse oral keratinocyte. [file 12964_2021_765_MOESM2_ESM.tif]

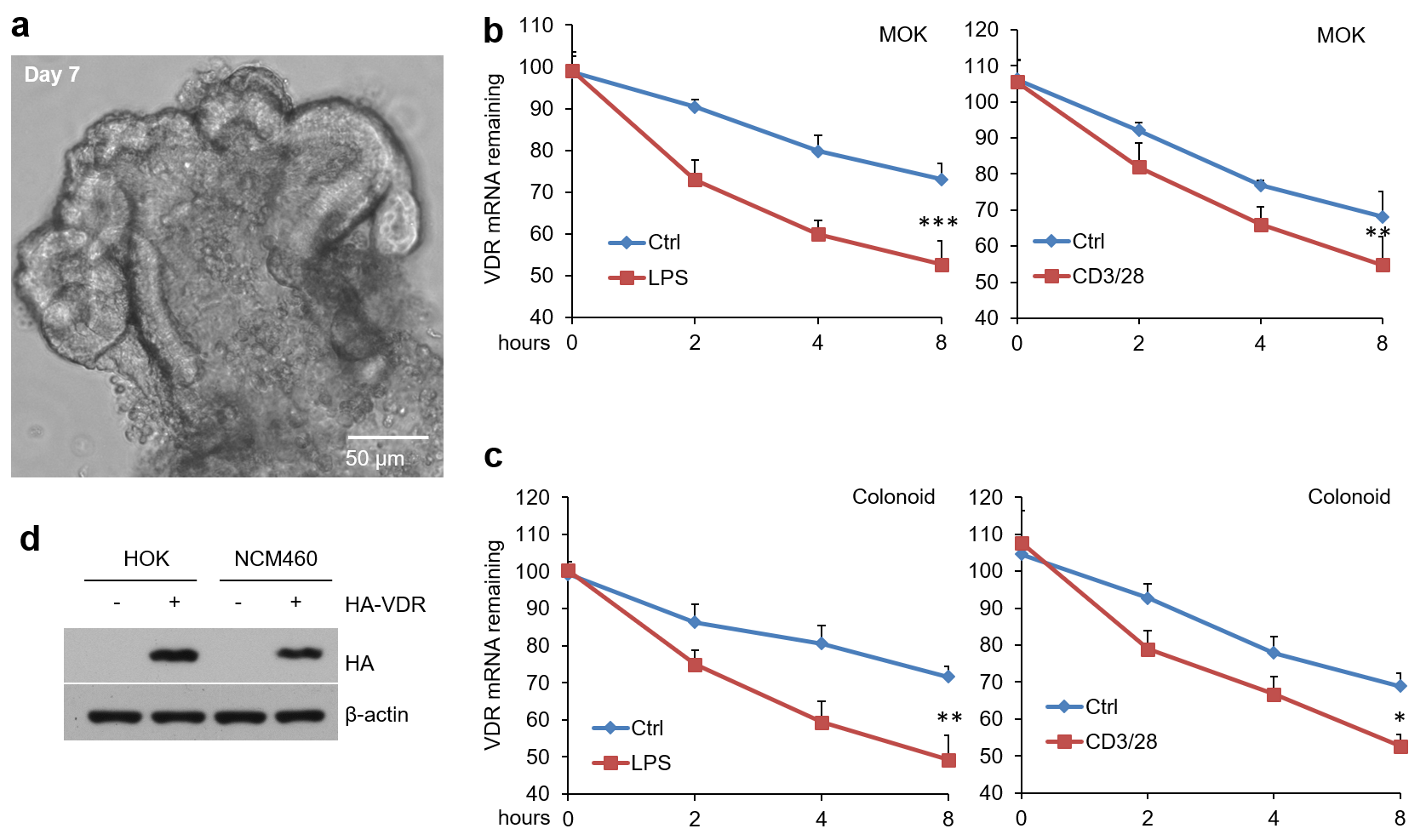

Supplement: Supplementary file 3 — Additional file 2.Supplemental figure 2. VDR mRNA degradation is observed in mouse oral and colonic epithelial cells following treatments. (a) Colonoid cultured from mouse colonic crypts. (b and c) VDR mRNA decay in MOKs (b) and colonoids (c) following LPS or activated CD4+ T cells treatment. (d) Western blot determinations of HOK and NCM460 cell lines transfected with HA-VDR plasmids. n = 3 each group, *P < 0.05, **P < 0.01, ***P < 0.001 versus corresponding control. MOK, mouse oral keratinocyte. [file 12964_2021_765_MOESM3_ESM.tif]

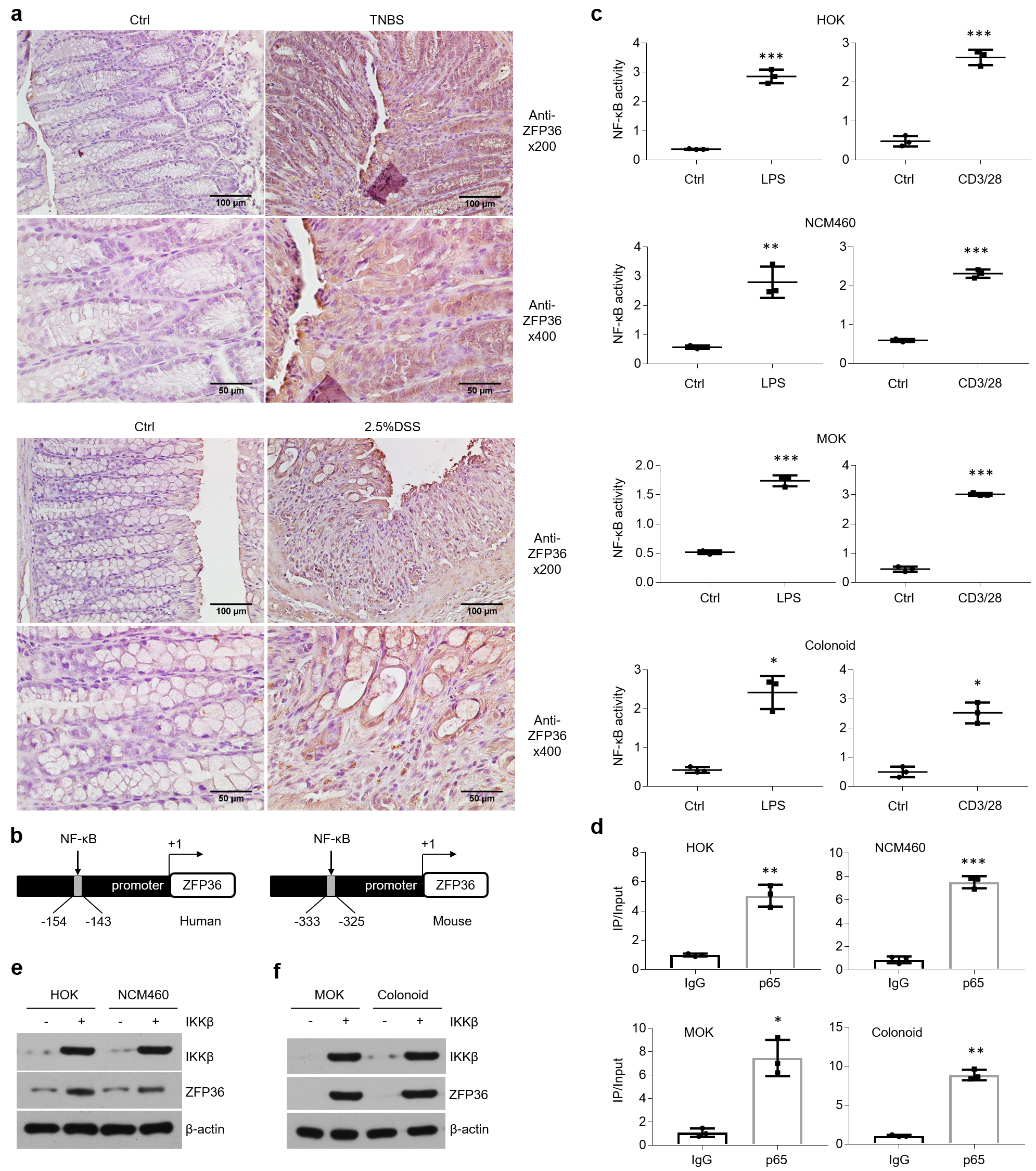

Supplement: Supplementary file 4 — Additional file 3.Supplemental figure 3. ZFP36 is induced by activated NF-κB pathway. (a) Immunostaining analyses of ZFP36 expression in the colonic mucosal tissues from TNBS- or DSS-treated mice. (b) Schematic illustration of NF-κB binding sites in the promoter region of ZFP36 gene. (c) NF-κB activity measurements in HOK, NCM460, MOK and colonoid following treatments as shown. (d) ChIP assays against NF-κB p65 antibody in epithelial cells as indicated. (e and f) Western blot detections of human (e) and mouse (f) epithelial cells with IKKβ plasmids transfection. n = 3 each group, *P < 0.05, **P < 0.01, ***P < 0.001 versus corresponding control. Ctrl, control; HOK, human oral keratinocyte; MOK, mouse oral keratinocyte. [file 12964_2021_765_MOESM4_ESM.tif]

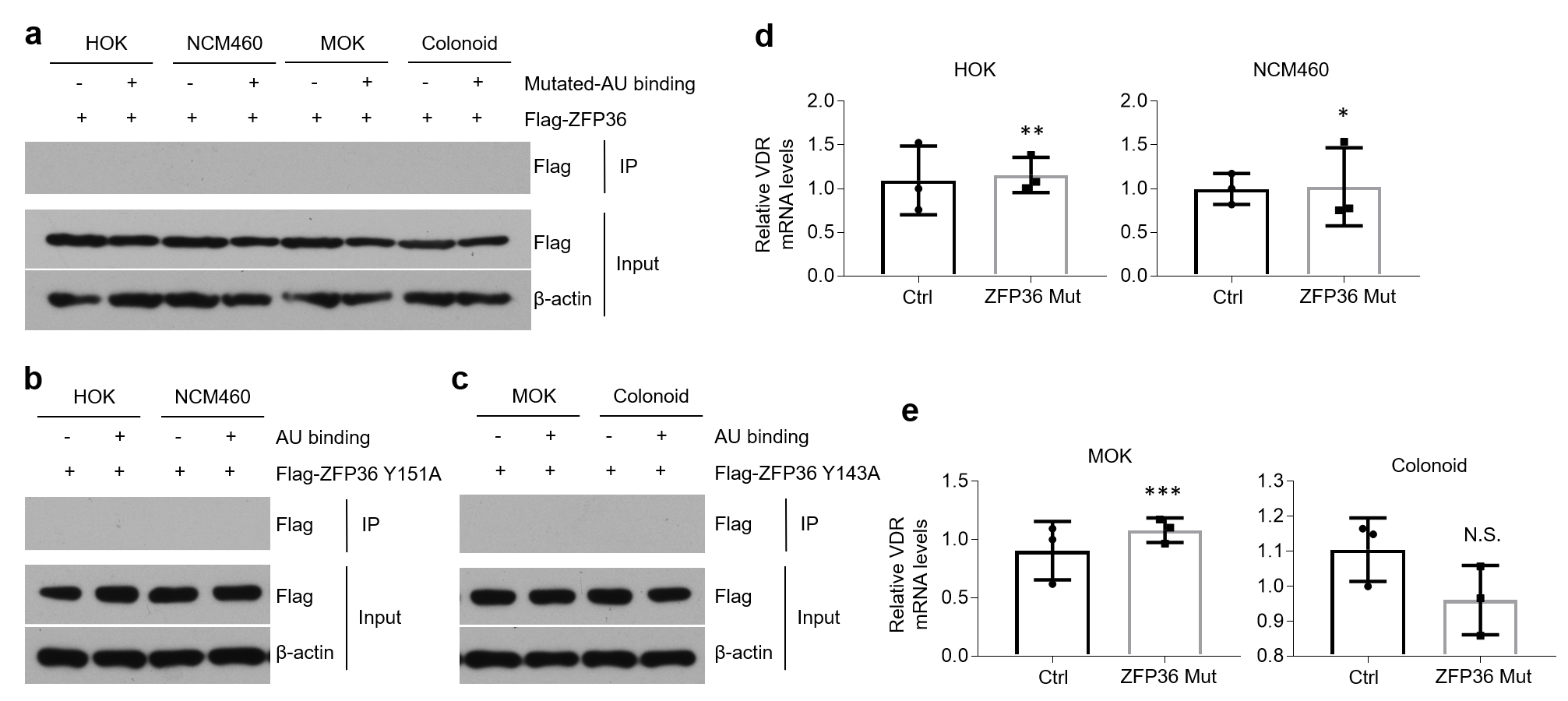

Supplement: Supplementary file 5 — Additional file 4.Supplemental figure 4. Mutated ZFP36 fails to bind with AREs in the 3’UTR of VDR mRNA. (a) Mutated-RNA probes pull-down and western blot examinations of cell lysates from cells transfected with ZFP36 plasmids. (b and c) RNA probes pull-down and western blot determinations of cell lysates from human (b) and mouse (c) cells transfected with ZFP36 mutated plasmids as shown. (d and e) Real-time PCR quantification of VDR mRNA levels in human (d) and mouse (e) cells transfected with ZFP36 mutated plasmids. n = 3 each group, *P < 0.05, **P < 0.01, ***P < 0.001 versus corresponding control. Ctrl, control; HOK, human oral keratinocyte; MOK, mouse oral keratinocyte. [file 12964_2021_765_MOESM5_ESM.tif]

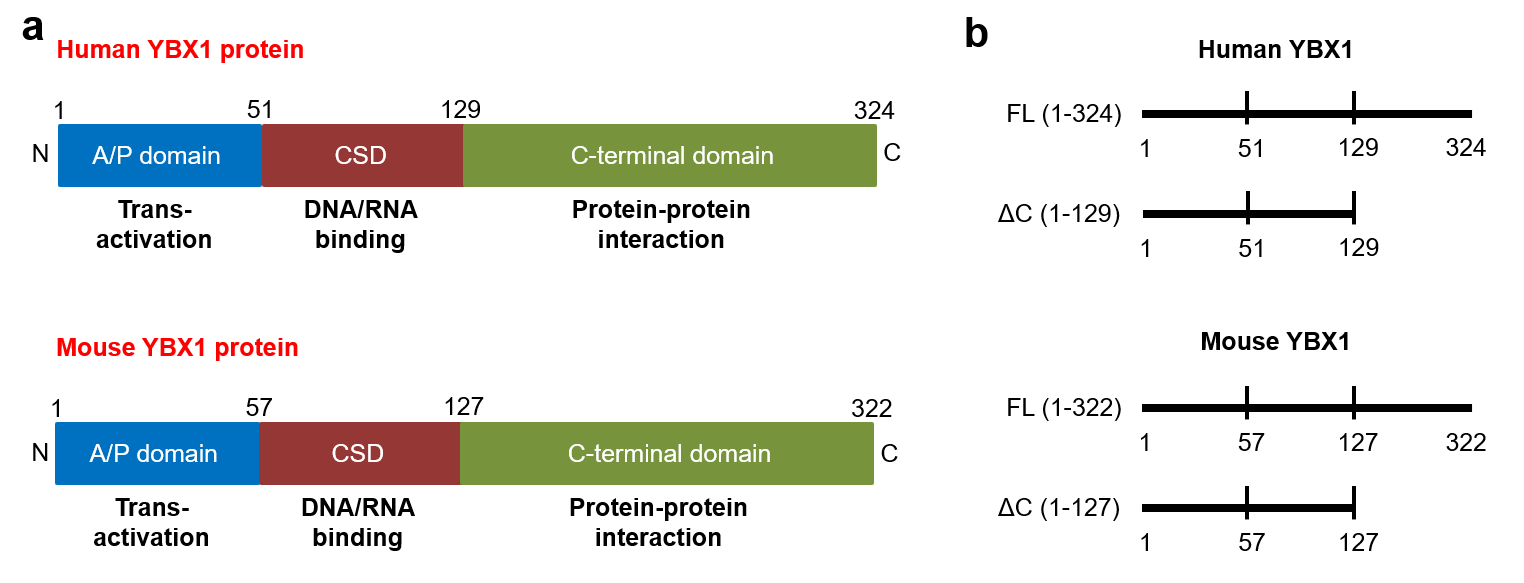

Supplement: Supplementary file 6 — Additional file 5.Supplemental figure 5. Construction of YBX-1ΔC plasmids. (a) Schematic structures of YBX-1 proteins. (b) The sketch map of plasmids. [file 12964_2021_765_MOESM6_ESM.tif]

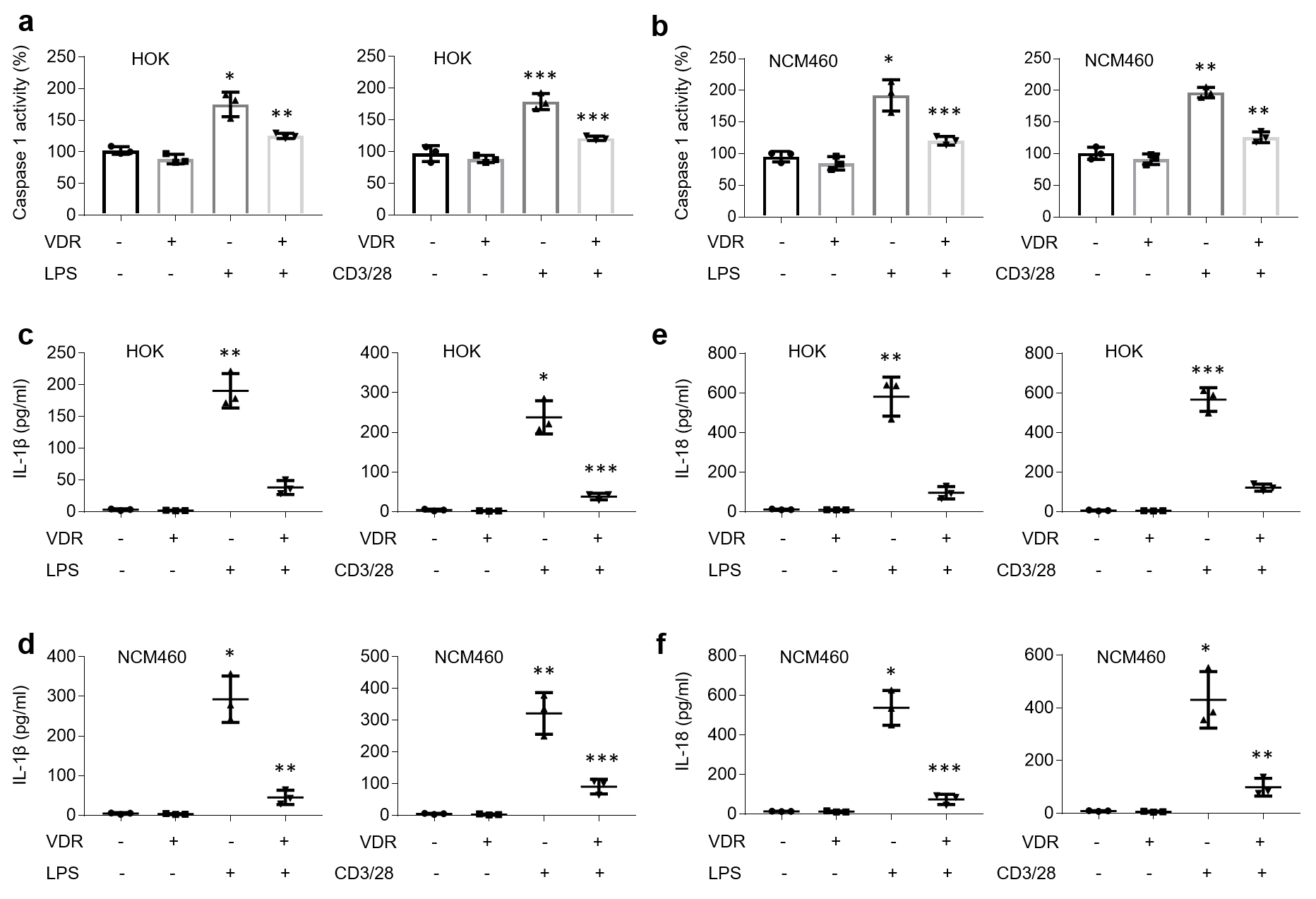

Supplement: Supplementary file 7 — Additional file 6.Supplemental figure 6. The enhanced caspase 1, IL-1β and IL-18 levels are relieved by VDR overexpression following treatments. (a and b) Caspase 1 activity assessments of HOK (a) and NCM460 (b) cell lines. (c and d) IL-1β concentrations in HOK (c) and NCM460 (d) cell lines. (e and f) IL-18 concentrations in HOK (e) and NCM460 (f) cell lines. Cells were transfected with empty or VDR plasmids following LPS or activated CD4+ T cells treatment. n = 3 each group, *P < 0.05, **P < 0.01, ***P < 0.001 versus corresponding control. HOK, human oral keratinocyte. [file 12964_2021_765_MOESM7_ESM.tif]
