# Supplementary material for: ZFP36 promotes VDR mRNA degradation to facilitate cell death in oral and colonic epithelial cells
Source: Cell Commun Signal. 2021 Aug 11;19:85. doi: 10.1186/s12964-021-00765-4 (PMC8355874; doi:10.1186/s12964-021-00765-4)
Supplement: Supplementary file 8 — Additional file 7. Primers and antibodies information. [file 12964_2021_765_MOESM8_ESM.docx]

**Supplemental table**

Supplemental table 1. Primer sequences involved in this study

| Primer name | Forward(5’-3’) | Reverse(5’-3’) |
| --- | --- | --- |
| hVDR | GACTTTGACCGGAACGTGCCC | CATCATGCCGATGTCCACACA |
| hZFP36 | GACTGAGCTATGTCGGACCTT | GAGTTCCGTCTTGTATTTGGGG |
| hGADPH | ACCACAGTCCATGCCATCAC | TCCACCACCCTGTTGCTGTA |
| mVDR | GATGCCCACCACAAGACCTA | CGGTTCCATCATGTCCAGTG |
| mZFP36 | TCTCTGCCATCTACGAGAGCC | TCCTCCGAGGGATTCGGTTC |
| mGADPH | TGTGTCCGTCGTGGATCTGA | CCTGCTTCACCACCTTCTTGA |
| hZFP36(ChIP) | GGCTGTCCACCGGCCAA | GCCGGCTACTTATAGGAAACTGC |
| mZFP36(ChIP) | CCTCAGTCTCTGCCCTTGTC | GGGGTAGTAGGGAGGGTTGT |
| hVDR plasmids | ATGGAGGCAATGGCGGCCAG | TCAGGAGATCTCATTGCCAAAC |
| mVDR plasmids | ATGGAGGCAATGGCAGCCA | TCAGGAGATCTCATTGCCGAAC |
| hZFP36 plasmids | ATGGATCTGACTGCCATCTA | TCACTCAGAAACAGAGATGC |
| mZFP36 plasmids | ATGGATCTCTCTGCCATCTA | TCACTCAGAGACAGAGATAC |
| hYBX-1 plasmids | ATGAGCAGCGAGGCCGAGAC | TTACTCAGCCCCGCCCTGCT |
| hYBX-1(ΔC) plasmids | ATGAGCAGCGAGGCCGAGAC | AGGACCTGTAACATTTGCTG |
| mYBX-1 plasmids | ATGAGCAGCGAGGCCGAGAC | TTACTCAGCCCCGCCCTGCT |
| mYBX-1(ΔC) plasmids | ATGAGCAGCGAGGCCGAGAC | AGGGCCTGTAACATTTGCTG |
| hVDR AU-rich | TGGCCCCATGTCTCTCAGAA | AGTGGTACCTGCTACCCTGTAT |
| mVDR AU-rich | AGAGGGCTAAGGTCAGACGG | TGGGAAAGCCATTCTCAGATGT |
| hVDR promoter  Luciferase report | ACAGGAATGAAATGAATGAAA | GCCAAGGCGCCCCGACAGAAGA |

Supplemental table 2. Antibodies information

| Antibodies | Source | Identifier |
| --- | --- | --- |
| VDR | Santa Cruz | Cat#: sc-13133 |
| β-actin | Santa Cruz | Cat#: sc-47778 |
| Anti-HA | Santa Cruz | Cat#: sc-7392 |
| Anti-NF-κB p65 | Cell Signaling | Cat#: 8242 |
| Anti-IKKβ | Cell Signaling | Cat#: 8943 |
| Anti-ZFP36 | ProteinTech | Cat#: 12737-1-AP |
| Anti-YBX-1 | ProteinTech | Cat#: 20339-1-AP |
| Anti-NLRP3 | Abcam | Cat#: ab214185 |
| Anti-Lamin C | Abcam | Cat#: Ab125679 |
| Anti-GAPDH | Sigma | Cat#: G8795 |
| Anti-Flag | MilliporeSigma | Cat#: F3165 |

**Supplemental figure legends**

Supplemental figure 1. VDR levels are decreased in mouse oral and colonic epithelial cells under inflammation condition. (a and b) Real-time PCR quantification of VDR in mouse primary oral (a) and colonic (b) epithelial cells with LPS or activated CD4+ T cells treatment, n = 3. (c and d) Western blot determinations of VDR levels in mouse primary oral and colonic epithelial cells following LPS (c) or activated CD4+ T cells (d) challenge, n = 3. (e and f) Schematic illustration of TNBS (e) or DSS (f) treatment protocol. (g) HE stained-colonic sections from control and TNBS- or DSS-treated mice. (h) Immunostaining showing VDR expression in the colonic epithelial cells of TNBS- or DSS-treated mice. **P* < 0.05, ***P* < 0.01, ****P* < 0.001 versus corresponding control. Ctrl, control; MOK, mouse oral keratinocyte.

Supplemental figure 2. *VDR* mRNA degradation is observed in mouse oral and colonic epithelial cells following treatments. (a) Colonoid cultured from mouse colonic crypts. (b and c) *VDR* mRNA decay in MOKs (b) and colonoids (c) following LPS or activated CD4+ T cells treatment. (d) Western blot determinations of HOK and NCM460 cell lines transfected with HA-VDR plasmids. n = 3 each group, **P* < 0.05, ***P* < 0.01, ****P* < 0.001 versus corresponding control. MOK, mouse oral keratinocyte.

Supplemental figure 3. ZFP36 is induced by activated NF-κB pathway. (a) Immunostaining analyses of ZFP36 expression in the colonic mucosal tissues from TNBS- or DSS-treated mice. (b) Schematic illustration of NF-κB binding sites in the promoter region of *ZFP36* gene. (c) NF-κB activity measurements in HOK, NCM460, MOK and colonoid following treatments as shown. (d) ChIP assays against NF-κB p65 antibody in epithelial cells as indicated. (e and f) Western blot detections of human (e) and mouse (f) epithelial cells with IKKβ plasmids transfection. n = 3 each group, **P* < 0.05, ***P* < 0.01, ****P* < 0.001 versus corresponding control. Ctrl, control; HOK, human oral keratinocyte; MOK, mouse oral keratinocyte.

Supplemental figure 4. Mutated ZFP36 fails to bind with AREs in the 3’UTR of *VDR* mRNA. (a) Mutated-RNA probes pull-down and western blot examinations of cell lysates from cells transfected with ZFP36 plasmids. (b and c) RNA probes pull-down and western blot determinations of cell lysates from human (b) and mouse (c) cells transfected with ZFP36 mutated plasmids as shown. (d and e) Real-time PCR quantification of *VDR* mRNA levels in human (d) and mouse (e) cells transfected with ZFP36 mutated plasmids. n = 3 each group, **P* < 0.05, ***P* < 0.01, ****P* < 0.001 versus corresponding control. Ctrl, control; HOK, human oral keratinocyte; MOK, mouse oral keratinocyte.

Supplemental figure 5. Construction of YBX-1ΔC plasmids. (a) Schematic structures of YBX-1 proteins. (b) The sketch map of plasmids.

Supplemental figure 6. The enhanced caspase 1, IL-1β and IL-18 levels are relieved by VDR overexpression following treatments. (a and b) Caspase 1 activity assessments of HOK (a) and NCM460 (b) cell lines. (c and d) IL-1β concentrations in HOK (c) and NCM460 (d) cell lines. (e and f) IL-18 concentrations in HOK (e) and NCM460 (f) cell lines. Cells were transfected with empty or VDR plasmids following LPS or activated CD4+ T cells treatment. n = 3 each group, **P* < 0.05, ***P* < 0.01, ****P* < 0.001 versus corresponding control. HOK, human oral keratinocyte.
